# Preliminary Study on the Development of a Real-Time Pressure-Monitoring Facial Mask for Burn Rehabilitation

**DOI:** 10.3390/ebj6010012

**Published:** 2025-03-03

**Authors:** Hyunjun Shin, Gyung-Jin Jeon, Seok-Jin Hwang, Hyeonseok Cho, Young-Min Cho, Hyoung-Soon Youn, Jisu Seo, Sehoon Park, Yoon-Soo Cho, Gyu-Seok Kim

**Affiliations:** 1 Korea Orthopedics & Rehabilitation Engineering Center, 10 Beon-gil, Gyeongin-ro, Bupyeong-gu, Incheon 21417, Republic of Korea; hyunjunshin5714@gmail.com (H.S.);; 2 T&L Co., Ltd. 767, Sinsu-ro, Suji-gu, Yongin-si 16827, Republic of Korea; 3 Burn Institute, Hangang Sacred Heart Hospital, College of Medicine, Hallym University, Seoul 07247, Republic of Korea

**Keywords:** severe burn, hypertrophic scar, pressure therapy, pressure mask, real-time pressure monitoring

## Abstract

The most common aftereffect of severe burns in patients is hypertrophic scarring. Hypertrophic scars typically form following severe burns; it refers to excessive collagen production in the dermal layer during the healing process, resulting in an abnormal raised scar. Currently, practical treatments for suppressing hypertrophic scars include laser therapy, pressure therapy, and the application of silicone sheets for moisture retention. The most extensively used treatment involves compression therapy using specially designed garments for the affected areas. However, this method has limitations when applied to curved surfaces like the face. To address this issue, three-dimensional (3D) scanning and 3D printing techniques have been actively developed for face masks and have shown promising clinical results. Unfortunately, current facial masks under development lack a sensor system to measure pressure, making it difficult to ensure consistent and appropriate pressures during clinical trials. In this study, we have developed a burn pressure mask capable of real-time pressure monitoring. The facial mask developed in this study utilizes an FSR-type sensor to measure the pressure applied to the skin. We have also embedded electrical wires within the mask to enhance its comfort and wearability. For this study, two patients wore the facial mask with real-time pressure measurement capabilities for 4 weeks in 12 h per day on average. We evaluated whether the mask maintained the appropriate pressure range (15–25 mmHg) throughout the clinical trial and whether it effectively inhibited scar formation. Through the analysis of recorded pressure signal data, we confirmed that the patients consistently maintained the appropriate pressure while wearing the mask during the clinical trial. Additionally, we observed significant differences in skin moisture levels, transepidermal water loss, and scar thickness before and after the experiment. These findings suggest that the facial mask, featuring real-time monitoring capabilities, effectively prevents the formation of hypertrophic scars.

## 1. Introduction

In South Korea, the number of burn patients resulting from workplace and household accidents totals approximately 500,000 to 600,000 annually. Among these cases, the number of severe burn patients requiring hospitalization consistently ranges from 20,000 to 30,000 (3–5%) [1]. In the United States, it is known that approximately 500,000 burn cases occur each year [2]. When comparing these figures relative to the population, South Korea has a burn patient incidence rate approximately six times higher than that of the United States [3]. This difference is attributed to the dietary habits of Koreans (who often enjoy hot soups), as well as to the structure of the Korean industrial sector, which is centered on manufacturing. As a result, South Korea has a relatively high number of burn patients per capita, underscoring the importance of burn rehabilitation treatments. In the case of severe burns (second-degree or higher), where both the endothelium and dermis are damaged, various complications and aftereffects can arise, with the most common being the development of hypertrophic scars [4,5]. These hypertrophic scars often are accompanied by symptoms such as skin protrusion, changes in skin color, itching, and discomfort [6,7,8,9]. In particular, because they are aesthetically unpleasing, it can significantly impact the patient’s psychological well-being, making it challenging to engage in normal social activities [10,11]. To mitigate these hypertrophic scars, various treatments have been developed, including pressure therapy using pressure garments, silicone-based products for moisture retention, and laser therapy [12,13]. Among these, pressure therapy, which is the most frequently employed, reduces hypertrophic scars by inhibiting collagen proliferation through the physical compression of the wound area, thereby reducing oxygen and blood flow [14]. According to Wei’s review paper, the pressure range of 15–25 mmHg is known to be the most effective, and most clinical trials employing pressure therapy aim to maintain this pressure range [15,16]. Currently, patient-fitted pressure garments are the most commonly used clinically, and have shown good clinical results [17,18,19,20,21]. However, pressure garments may have difficulty in providing uniform pressure to the affected area, particularly on the face, where the surface is highly curved [22]. To address these limitations, ongoing research actively explores the application of uniform pressure to the entire face through the use of three-dimensional (3D)-printed masks generated via noncontact 3D scanning [23]. Shons et al. reported that burn scarring was suppressed when patients wore a face mask for more than 20 h a day for 1.5 years in a study involving 97 patients over 6 years [24]. Wei et al. reported that scar thickness and hardness were significantly reduced when transparent 3D-printed masks were applied to 10 patients at a pressure of 13.32 mmHg [25]. A similar 2017 study by Wei reported comparable results in child burn patients [26]. Rivers et al. reported that wearing a mask that applied pressure of approximately 35 mmHg to the scar with an elastic strap for 20 h every day resulted in scar tissue shrinking and the suppression of hypertrophic scars [27]. Additionally, various studies have reported that pressure masks are producing good results in alleviating burn scars [15,26,28,29,30]. Most of these studies reported on the ability to adjust the mask pressure appropriately, but they did not include information on whether the pressure was consistently maintained during the clinical trials [31,32]. Given that both excessive and inadequate pressures can reduce the effectiveness of suppressing hypertrophic scars or lead to potential side effects, continuous monitoring of pressure and its maintenance within the desired range is indeed crucial for the success of these treatments [22].

In this study, we developed a burn facial mask equipped with an FSR-type pressure sensor for real-time pressure monitoring during clinical trials. Consistent with prior studies, we performed direct 3D scans of burn patients to create both facial and facial mask models, which were then used to produce the burn facial mask through 3D printing. In particular, our mask incorporated embedded wires (placed inside the mask to prevent exposure to the outside), ensuring both convenience and safety during daily use. The hypertrophic scar-suppressing effect of the burn mask was investigated using non-parametric statistical analyses before and after 4 weeks of wearing the mask on eight burn scars from two patients with facial burns. Additionally, we assessed whether the patients could maintain appropriate pressure by continuously monitoring it through a screen during the clinical trials.

## 2. Methods

### 2.1. Burn Mask Design and Manufacturing of Facial Masks

#### 2.1.1. 3D Scanning of Patient’s Face

**Figure 1:**
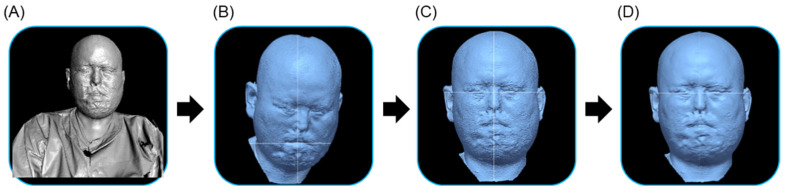
Process used to generate the facial model using three-dimensional (3D) scanning: (**A**) 3D scanning, (**B**) data filtering, (**C**) alignment of coordinates, and (**D**) surface smoothing within the 0.2–0.5 mm range.

To perform the 3D scanning of the burn patient’s face, we employed a noncontact handheld 3D light scanner (Artec Eva, Artec 3D, Luxembourg) while the patient was seated in a chair. The creation of the facial model involved a four-step process as shown in Figure 1, adhering to standard procedures in general reverse engineering for 3D model creation. These steps are as follows: (1) 3D scanning: noncontact optical scanning of the entire face to eliminate shading; (2) data filtering: integrating scanning values in a local coordinate system generated from various angles; (3) alignment of coordinates: integrating the facial model into a local coordinate system and converting it to a global coordinate system, and (4) surface smoothing: employing a process to eliminate irregular surfaces and achieve a smooth surface finish. The smoothing process was conducted by selecting a value within the 0.2–0.5 mm range that produced the least distortion or holes on the surface of the facial model.

#### 2.1.2. Generation of Facial Mask Model

**Figure 2:**
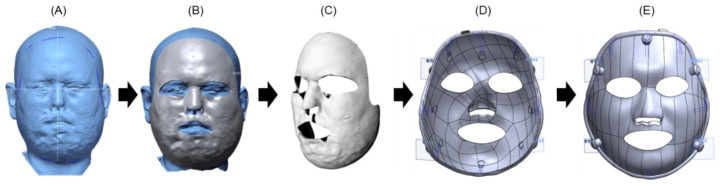
Generation of the facial mask model from the facial model. (**A**) Detection of the mask region from the patient’s head model (blue line), (**B**) creation of a surface model along the surface boundary, (**C**) conversion to a solid mask model from the surface model with a uniform thickness (2–3 mm), (**D**) generation of pressure sensor insertion regions and air holes for wire embedding, and (**E**) creation of hinges for attaching the strap.

Using the created facial model, we developed a facial mask model that could accommodate the insertion of a sensor as shown in Figure 2. The process began with discussions with the physician in charge to determine the mask’s size and outer boundaries, which were tailored to fit the patient’s facial shape and the location of the burn scar. Following the establishment of the outer boundaries by the physician, a mask surface model without thickness was created to match the patient’s face. Subsequently, a thickness ranging from 1 to 3 mm was set for the mask to reflect the use of a silicone sheet. An offset of 1 to 3 mm was added to the patient’s facial surface model to create a solid mask model with the required thickness. To reduce concentrated pressure on areas of the mask that interact with prominent bony structures, such as the eyelids and cheekbones, a slight offset of 0.5–1 mm was intentionally added to the mask design in these regions. Subsequently, we designed the mask to accommodate up to eight FSR sensors and consulted with the physician in charge to determine their placement near prominent scar locations where monitoring was most crucial. To facilitate the embedding of wires for transmitting power and signals from the FSR sensors, we included air holes inside the mask. Subsequently, we created five hinges on the surface of the mask where the straps could be attached, finalizing the mask model. The generation of both the facial and the facial mask models was accomplished using the Geomagic Design X 2020 software (Oqton, San Francisco, CA, USA). The sensor insertion points and airhole creation for the facial mask model were completed using the software Solidworks 2014 (Solidworks, Waltham, MA, USA).

#### 2.1.3. 3D Printing Production and Assembly of the Burn Facial Mask

**Figure 3:**
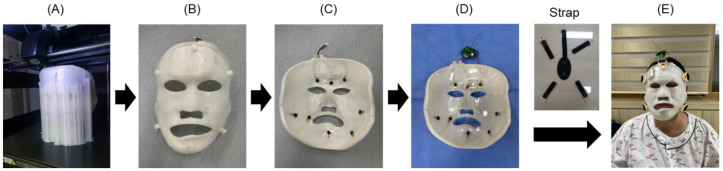
Manufacturing of the burn facial mask with an inner silicone layer and pressure sensor. (**A**) FDM-type 3D printing of the facial mask using PLA material, (**B**) facial mask prototype, (**C**) external facial mask embedding sensor, circuit, and connector, (**D**) attachment of silicone sheet onto the external mask, and (**E**) application of the facial mask with the strap to burn patient.

The 3D printing of the facial mask model was performed using the FDM method with a Style NEO-A31C (Cubicon, Sungnam-si, Republic of Korea) 3D printer with PLA filament (iPLA, Cubicon, transparent) as shown in Figure 3. Initially, facial masks were produced using the Polyjet method (which is a certified medical device method) using MED610 filament (Stratasys LTD, Rehovot, Israel), which is a transparent material type. However, patients expressed concerns about the visibility of the black dot-like FSR sensor, and the cost of Polyjet printing was relatively high. Consequently, we transitioned to the use of opaque PLA and cost-effective FDM printing. Up to eight pressure sensors can be inserted inside the mask, and their shape is shown in Figure 3D. Once the silicone sheet was firmly in place, a pressure control strap was attached to the hinge on the mask’s exterior. The mask was then ready to be worn by the patient.

### 2.2. Development of the Pressure-Sensing Module for Real-Time Measurements

**Figure 4:**
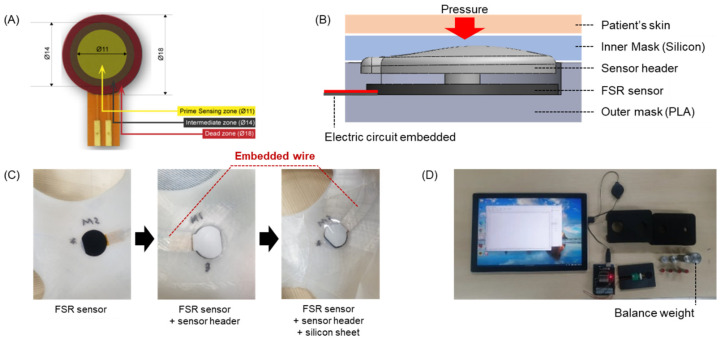
Pressure measurement sensor module design. (**A**) FSR sensor, (**B**) the structure of the sensor module, (**C**) the assembly of the sensor module, and (**D**) real-time pressure measurement setup with balance weight.

The pressure sensor used in the burn facial mask was the FSR type RA18 (Marveldex, Bucheon-si, Republic of Korea) (Figure 4A). As shown in Figure 4B, the pressure sensor is inserted into the sensor insertion groove inside the mask, and the sensor protection header and silicone inner skin sheet are attached in that order. As mentioned earlier, the power line and signal line connected to the sensor are embedded in the outer skin along the air hole for safety, and are connected to the integrated circuit at the top without being exposed to the outside. As the measured pressure itself is very small and both the silicone sheet and skin are soft materials, there is a problem with pressure transmission (pressure is smaller). Therefore, the sensor contact surface within the shell is designed to contain protrusions to concentrate the pressure, enabling the sensor to measure pressure sensitively. In the case of the FSR sensor, because the signal value responds sensitively to shear forces, the sensor header was manufactured to fit as closely as possible to the sensor insertion part of the outer shell to minimize pressure errors due to shear. The final sensor assembly is depicted in Figure 4C. Figure 4D illustrates the setup for sensor signal measurement, including the manufactured module and monitoring device. In this study, an 8-channel ADC board was developed using a low-power controller (STM32L432) and a Bluetooth communication module to monitor multiple pressure sensor values simultaneously. The measurement board was affixed to a strap above the clinical patient’s head; accordingly, pressure values measured by the sensor could be monitored through Bluetooth communication with a tablet.

### 2.3. Development of the Strap System

**Figure 5:**
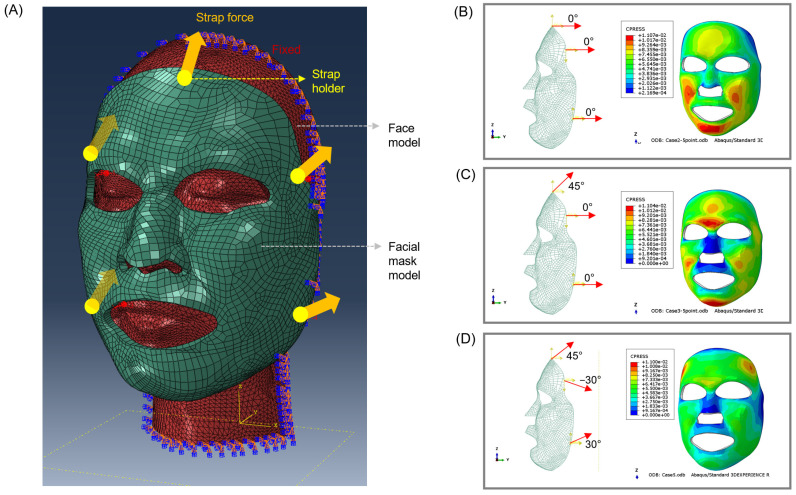
Finite element analysis of facial mask model. (**A**) Facial mask model used for finite element analyses, (**B**) uniform pressure distribution (parallel axis), (**C**) pressure distribution at 45° with top position, and (**D**) pressure distribution at 45° with respect to the top position and 30° with respect to the side position.

The pressure in the affected area is primarily influenced by the direction of the strap and the applied force. In this study, an FEM model was developed, as depicted in Figure 5A, to determine the optimal force direction for generating uniform pressure while wearing the mask. The analysis model was intentionally simplified to focus on the identification of the strap direction that would result in the most uniform pressure distribution. To create the head dummy model, the entire head of a randomly selected subject (male, age: 50 years) was scanned. The mask model was designed using Geomagic Design. The internal surface area of the mask model, determined through a design program, was 40,000 mm^2^. When a force of 133 N was applied perpendicular to the internal surface, it generated a pressure of 25 mmHg (3333.05 Pa). As a result, the force in the *y*-axis direction was evenly distributed, with each of the five strap points withstanding forces equal to 26.6 N. To simplify the analysis, we treated the head model as a rigid body, and we assumed a frictionless interface between the skin and the mask. As shown in Figure 5D, the analysis revealed that the most uniform pressure was achieved when the strap was pulled at a 45° angle for the upper part, −30° for the upper strap, and +30° for the lower strap. Accordingly, the strap was designed to reflect these analysis results. Furthermore, we designed the strap to allow for incremental adjustments using a boa system. Two straps were affixed to the upper and lower regions at the back of the head to facilitate partial pressure adjustments.

### 2.4. Clinical Test Method

The clinical trial involved two adult patients who had sustained deep second-degree or more burns on their faces and were undergoing burn rehabilitation treatment after reaching the epithelialization stage through aseptic wound treatment or skin graft surgery. Details of the subjects are summarized in Table 1. A facial mask was produced for each patient using the method outlined in Section 2.1. Prior to the 4-week clinical trial, both patients wore the masks for one week to confirm the absence of side effects. This study was approved by the institutional review board of The Hangang Sacred Heart Hospital (HG 2023-014), and written informed consent was obtained from all study subjects who voluntarily participated. This study was registered on the Clinical Research Information Service (KCT0005918). Before commencing the clinical trial, the calibration of the pressure sensor measurement module was verified using an external pressure gauge to ensure the appropriate pressure was generated. In this study, two FSR sensors were inserted into the left and right cheek areas to monitor the actual pressure values during the periods the masks were worn. These sensors allowed the patients to adjust the strap while wearing the mask to maintain the desired pressure range of 15–25 mmHg. Pressure measurements were recorded to confirm the maintenance of appropriate pressure during the clinical trials. The clinical trial results were assessed both quantitatively and qualitatively. The quantitative evaluation included five parameters: skin water content, transepidermal water loss, melanin, erythema, and scar thickness. Measurements for skin water content, transepidermal water loss, melanin, and erythema were conducted using a Tewameter, Corneometer, and Mexameter (Multiprobe adapter, Courage + Khazaka Electronic GmbH, Köln, Germany), respectively. Scar thickness was measured using E-CUBE 7 (Alpinion Medical Systems, Seoul, Republic of Korea). Eight scars in total, four for each patient, were assessed to determine if there was a significant difference before and after the masks were worn. Statistical analysis for quantitative evaluation was performed using the Wilcoxon signed-rank test with the software GraphPad Prism (version 9, San Diego, CA, USA). The Wilcoxon signed-rank test was applied to compare pre- and post-treatment data due to the non-normal distribution of the variables and the paired nature of the measurements. Qualitative evaluations included assessments based on the Vancouver Scar Scale (VSS) and the Patient and Observer Scar Assessment Scale (POSAS). These assessments were conducted based on questionnaire responses and covered five VSS items, six observer scale items of POSAS, and seven patient scale items of POSAS.

## 3. Results

### 3.1. Sensor Calibration and Monitoring

#### 3.1.1. Sensor Grouping

**Figure 6:**
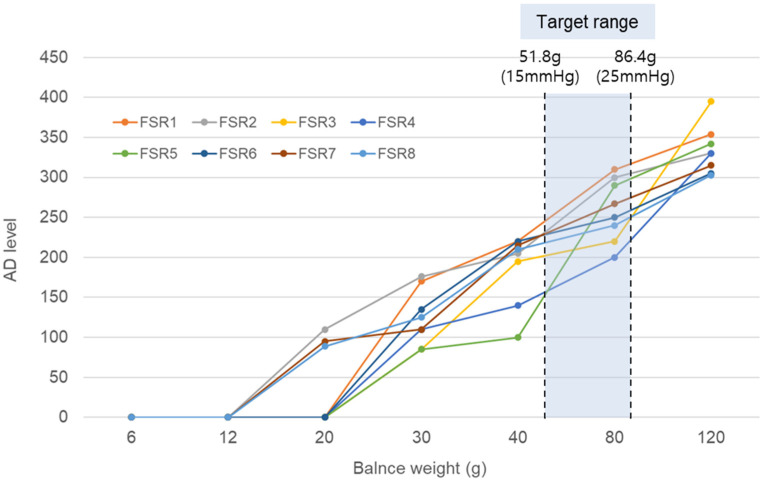
Calibration of all FSR sensors. The region highlighted in blue represents the target pressure range.

Each FSR sensor exhibits significant variations in sensitivity to pressure. Thus, selecting sensors with comparable sensitivities is essential to ensure the reliability of a real-time pressure measurement mask. Combining sensors with markedly different sensitivities may lead to situations where some sensors register pressure while others do not detect any pressure at all. In this study, sensor grouping was performed by applying weights based on sensitivity (ranging from 0 g to 120 g to the sensor modules) and measuring the analog-to-digital (AD) levels to record the corresponding voltage values according to the applied pressure. Figure 6 presents the AD levels obtained for the weights of 6 g, 12 g, 20 g, 30 g, 40 g, 80 g, and 120 g in the cases of the eight tested sensors. As this experiment focused on the observation of sensor tendencies, precise voltage values were not essential. Taking into consideration the size of the FSR sensor, the weight corresponding to a pressure of 15 mmHg was 51.8 g, while the corresponding weight for a pressure of 25 mmHg was 86.4 g. Consequently, only the sensors that exhibited similar trends in response to the weights of 40 g and 80 g were collected and grouped. For example, as shown in Figure 6, sensors FSR 1, 2, 6, 7, and 8, were grouped, excluding FSR 3, 4, and 5.

#### 3.1.2. Simultaneous Pressure Measurement Results

**Figure 7:**
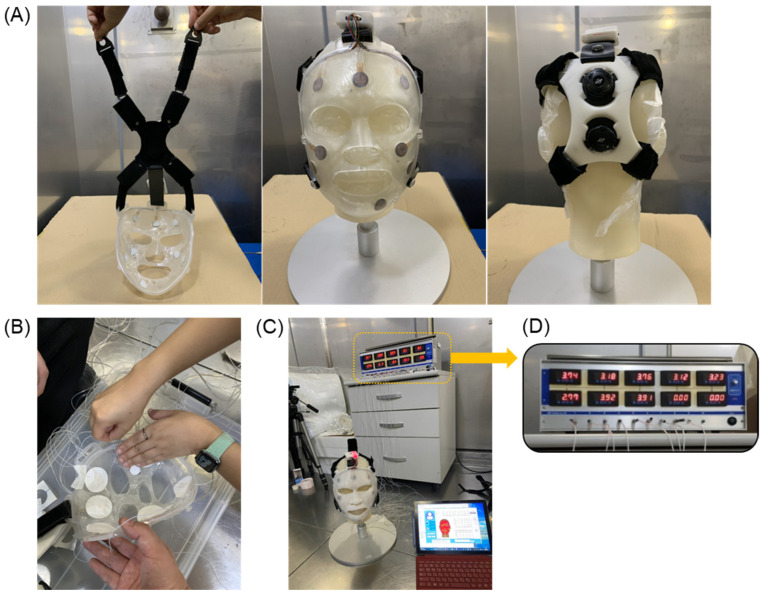
Real-time measurement test with eight FSR sensors. (**A**) Dummy model for real-time measurement, (**B**) airbag-type sensor with facial mask, (**C**) setup of pressure measurement system, and (**D**) real-time measurement results of eight FSR sensors.

Given the differing sensitivities of each FSR sensor, it was important to assess whether the strap adjustment could apply suitable pressure to all eight sensors inside the mask simultaneously. This aspect was also examined during the sensor grouping process described in Section 3.1.1. For this evaluation, real-time pressure measurements were performed using the contact pressure measuring device, AMI-3037-10 (AMI Techno, Tokyo, Japan). The pressure unit for the AMI-3037-10 device was in kPa, and the equivalent values for 15 and 25 mmHg were 1.1 and 3.2 kPa, respectively. First, a head dummy model designed to resemble the patient’s head was created, as shown in Figure 7A, and a facial mask with built-in pressure sensors was fitted onto it. An airbag-type pressure measurement sensor was placed between the mask and the dummy, as depicted in Figure 7B. As the strap was adjusted to regulate the pressure on the eight sensors, it was confirmed that all of the sensors maintained an appropriate pressure within the range of 15–25 mmHg simultaneously, as illustrated in Figure 7D. These results indicate that the strap can be tailored to ensure uniform and suitable pressure is exerted on all eight sensors simultaneously, without exceeding the specified bounds.

#### 3.1.3. Pressure Sensor Calibration with Air Cell Measurement System Before Clinical Test

**Figure 8:**
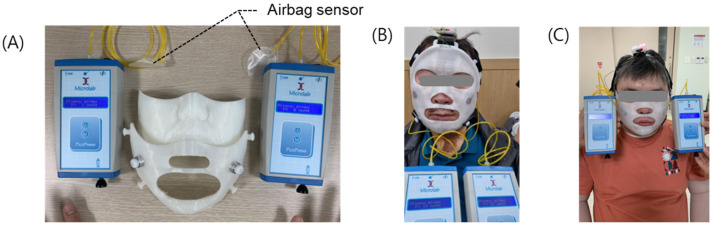
Initial sensor calibration before the onset of the clinical test. (**A**) The PicoPress setup for sensor calibration for (**B**) subject 1 and (**C**) subject 2.

Calibration of the final pressure mask sensor was performed for each patient using the PicoPress equipment (Microlab, Padua, Italy) before the onset of clinical trials as shown in Figure 8. The PicoPress airbag patch was attached to the patient’s skin, the mask was worn, and the straps were adjusted to measure the actual pressure of the PicoPress on the skin. The ADC level of the sensor was measured when the PicoPress pressure values were 15 and 25 mmHg and linearized to a first-order linear regression. During clinical trials, the pressure value applied to the skin was inferred by interpolating this regression line as a base. Based on this calibration process, the pressure value induced on the actual skin and the voltage value measured by the sensor were matched.

### 3.2. Clinical Tests

#### 3.2.1. Daily Pressure Measurements

**Figure 9:**
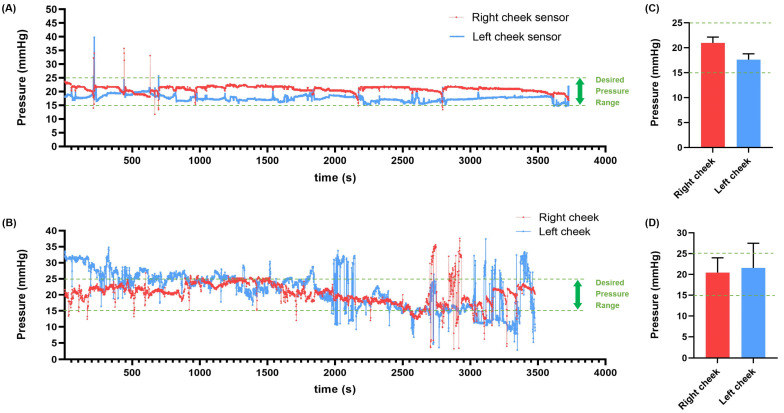
Real-time pressure measurements during the first 1 h of the clinical trial. Overall pressure graphs of (**A**) Subjects 1 and (**B**) Subjects 2, Average pressure values of (**C**) Subjects 1 and (**D**) Subjects 2 (Green line: desired pressure range, 15–25 mmHg).

In the case of burn pressure treatment, it is crucial to maintain a consistent pressure within the range of 15–25 mmHg for an extended period. The research team requested that the two clinical patients monitor and maintain pressures in the range of 15–25 mmHg throughout the duration for which the masks were worn. The pressure applied to the patients during the clinical trials was recorded on an SD card, and the research team collected these recorded pressure values during all of the patients’ treatment visits. Figure 9 presents a part of the pressure measurement values recorded during the actual clinical trials. Figure 9A,C displays the monitored values for the initial one hour of wear for each patient, while Figure 9B,D illustrates the average pressure values and standard deviations over the entire period. Figure 9 shows that the patients monitored the pressure in real-time and successfully maintained an average pressure value within the 15–25 mmHg range throughout the clinical trial period by adjusting the straps as needed.

#### 3.2.2. Clinical Test Results

**Figure 10:**
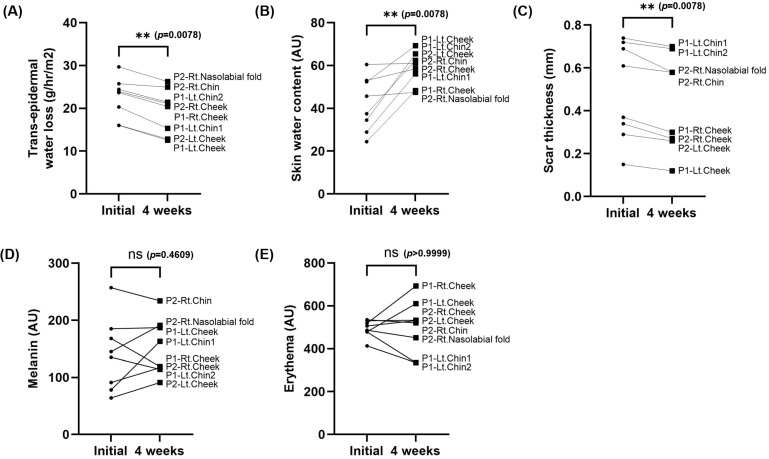
Comparison of changes in scar biomechanical properties between the initial and 4 weeks group. (**A**) Transepidermal water loss (*p* = 0.0078), (**B**) skin water content (*p* = 0.0078), (**C**) scar thickness (*p* = 0.0078), (**D**) melanin (*p* = 0.4609), and (**E**) erythema (*p* > 0.9999), (** *p* < 0.01).

Figure 10 presents the statistical results following 4 weeks of clinical testing. The symbol P1 corresponds to patient 1, while P2 represents patient 2. This study examined a total of eight burn scar areas, with four areas evaluated for each patient. Statistical analysis of the 4-week compression therapy demonstrated a significant reduction in epidermal evaporation rate and scar thickness, along with an increase in moisture content. Both the reduction in the epidermal moisture evaporation rate and the increase in moisture content are believed to be primarily attributed to the silicone sheet’s application, as reported in previous studies. The reduction in scar thickness was also attributed to the effect of the applied pressure. However, melanin and edema did not exhibit significant trends. This is likely because melanin and edema require an extended period to exhibit noticeable improvements. The limited duration of mask-wearing during this study may have contributed to the fact that these factors did not exhibit significant changes.

**Figure 11:**
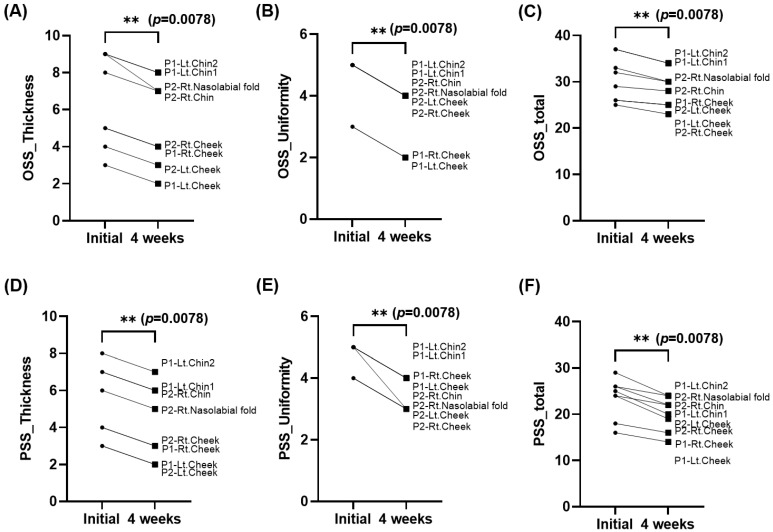
Comparison of changes in the Vancouver Scar Scale (VSS) and the Patient and Observer Scar Assessment Scale (POSAS) between the initial and 4-week groups. Since all VSS parameters were statistically non-significant, they have been excluded from the analysis. Among the POSAS parameters, only the statistically significant results are presented: (**A**) POSAS_Observer_Thickness (*p* = 0.0078), (**B**) POSAS_Observer_Uniformity (*p* = 0.0078), (**C**) POSAS_Observer_Total (*p* = 0.0078), (**D**) POSAS_Patient_Thickness (*p* = 0.0078), (**E**) POSAS_Patient_Uniformity (*p* = 0.0078), and (**F**) POSAS_Patient_Total (*p* = 0.0078). The remaining POSAS parameters were excluded due to lack of statistical significance (** *p* < 0.01).

The qualitative research results are presented in Figure 11. The VSS scar scale did not yield any significant changes in either the individual scores or the total score. It is believed that the lack of significant changes in each item was due to the relatively short clinical observation period. Regarding the observer scale of the POSAS, the survey results indicated that the pressure mask was effective in reducing scar thickness and promoting scar surface uniformity. These findings are consistent with the observed quantitative measurement trends. There were no notable differences in other aspects. The patient scale of the POSAS yielded the most positive survey results for scar thickness and uniformity. This alignment with both the quantitative measurements and patient perceptions suggests that the pressure mask can significantly reduce scars. Given the positive quantitative and qualitative results, and the confirmation of its feasibility in the short-term study, it is imperative to further evaluate its performance through a long-term study in the future.

## 4. Discussion and Conclusions

**Figure 12:**
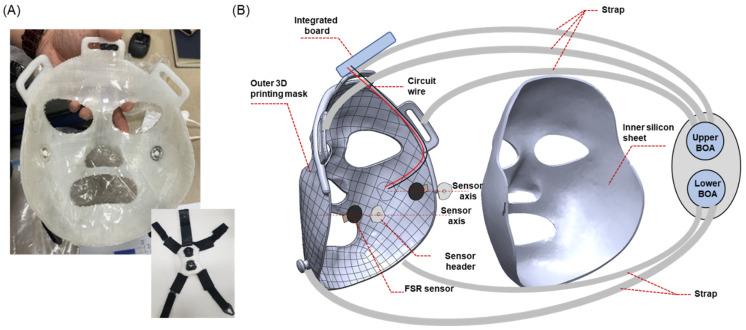
(**A**) Prototype of burn facial mask, and (**B**) sectional view of the burn facial mask.

The goal of this study was to develop a pressure-monitoring mask to inhibit the growth of hypertrophic scars in patients with burns and to demonstrate the preliminary therapeutic effect of the mask through clinical trials. To treat hypertrophic scars, researchers have focused on creating and improving a custom 3D compression mask that incorporates pressure sensors, which enable the measurement of contact pressure within the safe range of 15–25 mmHg. Effective compression around burn scars is vital, as it helps to inhibit myofibroblast activity, limit the oxygen and nutrient supply to the affected area, and influence collagen production [33,34]. This is important because pressures above 30–40 mmHg can result in harmful effects, including reduced venous return, sleep apnea, and disruptions in autonomic nervous system function [25,27,35,36]. Differing from previous burn masks, the pressure mask developed in this study offers the advantage of real-time pressure monitoring through the integration of FSR sensors. In addition, the mask’s thickness allows the wires and electrical circuits connected to the sensor to be embedded into the outer layer, making it more convenient to wear without causing discomfort, as shown in Figure 12. The sensor used in this study underwent a two-step calibration to accurately measure the pressure induced on the skin. First, because the sensitivity of the FSR sensor was different for each sensor, we grouped sensors with similar sensitivities using weights and assembled them into one mask. Furthermore, because the ad level measurement results of the sensor measured using weights cannot reflect differences in the actual patient’s skin stiffness or silicone adhesion, the final sensor calibration was performed for each patient using the PicoPress system. Based on the calibration described above, accurate measurements of the pressure induced on the patient’s skin became possible using the FSR sensor. For reference, the force value measured using a weight with a hard surface tended to be somewhat larger than the pressure measured on the skin using the PicoPress system. This is thought to reflect the characteristics of hard materials.

A clinical trial was conducted with two patients who had facial burns. Before the trial, individual FSR sensors were carefully calibrated using the PicoPress system and integrated into the monitoring program. Throughout the clinical period, the patients diligently monitored pressure values and made efforts to maintain the appropriate pressure, as required. The results of these efforts are illustrated in Graph 10. As depicted in Figure 9, it was evident that the measured pressure varied owing to various factors such as breathing, various facial expressions, and changes in posture. Patients made adjustments by tightening or loosening the straps to maintain the desired pressure. Patients indicated that they felt confident about their treatment progress by confirming that pressure was consistently maintained at optimal levels through the monitored values. Furthermore, by reviewing weekly monitoring data for each patient, the medical staff and the development team could assess the total wearing time and the pressure levels during wear, which contributed to their confidence in the efficacy of burn compression treatment. The clinical trial yielded quantitative results that showed significant differences in terms of moisture content, epidermal moisture evaporation rates, and scar thickness as a result of using the silicone sheet and continuous pressure. Notably, patients reported a perceived reduction in scar thickness. The increase in skin hydration and the reduction in scar thickness are likely due to the combined effects of three factors: the consistent application of an optimal pressure range, the use of silicone material, and the enhanced adhesion of the 3D mask to facial scars, which ensures better contact with all facial contours. These early clinical results suggest the potential effectiveness of the pressure mask in suppressing hypertrophic scarring. However, no substantial differences were observed in indicators like melanin and erythema. It is believed that these indicators may exhibit improvements through extended periods of compression treatment. Patients also reported minimal discomfort, pain, or itching during prolonged wear, emphasizing the product’s suitability for everyday use. Nonetheless, owing to direct contact with the skin, the inner silicone layer accumulates foreign substances, necessitating further development to facilitate easier cleaning. Additionally, it was noted that the sensor-embedded mask posed cleaning challenges. To address this, future iterations should consider incorporating a waterproof feature to eliminate cleaning issues. Furthermore, for a comprehensive study on the effectiveness of real-time pressure monitoring, it is recommended to include results from patients wearing masks (without monitoring) as an intermediate control group. Future research plans will encompass a broader examination scope involving these groups. Lastly, pressure masks are designed and manufactured anew every 2–3 months to accommodate changes in scar shape. Streamlining the manufacturing process is crucial, as the entire production cycle must be repeated during each replacement. Currently, it takes approximately 1 week to produce the pressure mask, with a plan to reduce the production period to around 3–4 days by simplifying the manufacturing process in the future.

## Figures and Tables

**Table 1:** Subject information of participants in the clinical tests.

	Subject 1	Subject 2
Gender	Male	Male
Age (years)	62	41
Burn type	Flame burn (FB)	Flame burn (FB)
Burn lesions without and with mask	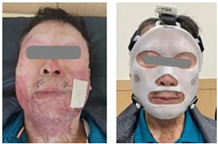	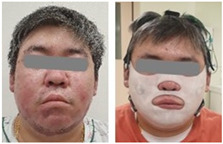
Number of sensors	2	2
Test period	4 weeks	4 weeks
Wearing time	12 h per day on average	12 h per day on average
Evaluation	(Quantitative evaluation) skin water content, transepidermal water loss, melanin, erythema, and scar thickness. Qualitative evaluation: Vancouver Scar Scale (VSS) (five parameters) and Patient and Observer Scar Assessment Scale (POSAS) (observer scale: six parameters; patient scale: seven parameters).	

## Data Availability

Data are contained within the article.
